# 

**Published:** 1857-12

**Authors:** 


### ELEVENTH VOLUME

OF THE

### DENTAL REGISTER OF THE WEST.

EDITED BY

**J. TAFT and G. WATT.**

TERMS: - - - - - - - $3 PER ANNUM, IN ADVANCE.

### Advertisements Inserted as follows:

One page, per annum,.......................................$25 00

One-half page, per annum........................................ 15 00

Quarter page, per annum,........................................ 10 00

Shorter periods than one year, by special arragement.

☞Contributions to the pages of the *Register* respectfully solicited.

Exchanges and Correspondents will please address "Dental Register," or "Taft & Watt, Cincinnati, Ohio."

